# Cerebrospinal fluid interleukin-6 is a potential diagnostic biomarker for central nervous system involvement in adult acute myeloid leukemia

**DOI:** 10.3389/fonc.2022.1013781

**Published:** 2022-12-01

**Authors:** Jiayan Gu, Xin Huang, Yi Zhang, Chenhui Bao, Ziyang Zhou, Hongyan Tong, Jie Jin

**Affiliations:** ^1^ Institute of Hematology, Department of Hematology, the First Affiliated Hospital, College of Medicine, Zhejiang University, Hangzhou, China; ^2^ Key Laboratory of Hematologic Malignancies, Diagnosis and Treatment, Zhejiang, Hangzhou, China

**Keywords:** cerebrospinal fluid, interleukin-6, central nervous system, acute myeloid leukemia, biomarker

## Abstract

**Objective:**

We evaluated the correlation between cerebrospinal fluid (CSF) cytokine levels and central nervous system (CNS) involvement in adult acute myeloid leukemia (AML).

**Methods:**

The study sample consisted of 90 patients diagnosed with AML and 20 with unrelated CNS involvement. The AML group was divided into two sub-groups: those with (CNS+, n=30) and without CNS involvement (CNS-, n=60). We used a cytometric bead assay to measure CSF interleukin (IL)-2, IL-4, IL-6, and IL-10, tumor necrosis factor-α, interferon-γ, and IL-17A. We used receiver operating characteristic curves to evaluate the ability of CSF cytokine levels to identify CNS involvement in adult AML.

**Results:**

CSF IL-6 levels were significantly higher in CNS+adult AML patients and positively correlated with the lactate dehydrogenase levels (r=0.738, p<0.001) and white blood cell (WBC) count (r=0.455, p=0.012) in the blood, and the protein (r=0.686, p<0.001) as well as WBC count in the CSF (r=0.427, p=0.019). Using a CSF IL-6 cut-off value of 8.27 pg/ml yielded a diagnostic sensitivity and specificity was 80.00% and 88.46%, respectively (AUC, 0.8923; 95% CI, 0.8168–0.9678). After treating a subset of tested patients, their CSF IL-6 levels decreased. Consequently, the elevated CSF IL-6 levels remaining in CNS+ adult AML patients post-treatment were associated with disease progression.

**Conclusion:**

CSF IL-6 is a promising marker for the diagnosis of adult AML with CNS involvement and a crucial dynamic indicator for therapeutic response.

## Introduction

Central nervous system (CNS) involvement is a well-known and serious extramedullary disease in adults with acute myeloid leukemia (AML). However, it is less common than acute lymphoblastic leukemia (ALL) (1–3). The therapies available for ALL and AML have significantly improved, and viable CNS disease treatments, such as intrathecal chemotherapy and more refined radiation techniques, have been developed. Despite this progress, CNS relapses remain life-threatening clinical and therapeutic challenges (4, 5). Most studies have focused on CNS involvement in pediatric AML patients; additionally, routine diagnostic lumbar punctures (LPs) are not performed in adult AML patients without obvious CNS signs or symptoms. Consequently, little is known about the incidence and clinical course of in adult AMLwith CNS involvement. This lack of information often results in unfavorable outcomes (5–7).

The current diagnosis of CNS diseases includes the clinical evaluation of neurologic symptoms and cerebrospinal fluid (CSF) by LPs, as well as radiographic imaging, which may be used independently or in combination (4). The LPs and conventional cytological examination of CSF is the standard diagnostic method for CNS leukemia (8). Recent studies have shown that flow cytometry (FCM) has superior specificity and sensitivity in detecting CNS leukemia compared to conventional cytology (CC) (9–11). However, both ways yield a high percentage (>40%) of false-negative reports owing to the low cellularity in the CSF. This percentage of false-negative reports was obtained from patients who were eventually diagnosed with CNS involvement by neuroimaging or autopsy (12). In addition, clinical evaluation of CNS leukemia may overlap with other neurological conditions, and radiographic imaging is not routinely performed during AML and ALL diagnosis, especially when there are no clinical symptoms (4, 13). Thus, developing effective diagnostic tools to detect CNS involvement in acute leukemia is imperative.

CSF biomolecules can be used as biomarkers to promote the diagnosis of CNS disease (14–16). Epstein-Barr viral DNA and the germ cell markers, α-fetoprotein and/or human chorionic gonadotropin, are applied in the diagnosis of AIDS-related CNS lymphoma and childhood CNS germinoma, respectively, and the differential expression of microRNAs in the CSF are potential noninvasive biomarkers for the diagnosis of CNS lymphoma (17–19). In primary central nervous system lymphoma (PCNSL), CSF levels of interleukin (IL)-10 have been reported as potential diagnostic and prognostic biomarkers (20, 21). Meanwhile, in large B cell PCNSL, increased IL-10/IL-6 ratios have been reported to be reliable diagnostic biomarkers (22). Some cytokines and chemokines, such as C-X-C motif chemokine ligand 10 (CXCL10), C-C motif chemokine ligand 4 (CCL4), CCL17, and IL-8, are also highly expressed in the CSF of metastatic tumors (23). In addition, a recent study reported that CSF miR-181a quantification might provide novel tools for monitoring CNS involvement in pediatric ALL (24). However, CSF biomarkers for the diagnosis of CNS involvement in adult AML patients have rarely been reported.

Thus, in this study, we retrospectively analyzed cytokine expression in the CSF of adult AML patients at CNS relapse. We explored the correlation between CSF cytokine levels and clinical characteristics of CNS involvement in these patients. We evaluated the diagnostic significance of CSF IL-6 and examined the cytokine levels after treatment to evaluate the dynamics of treatment-induced alteration of the IL-6 expression. Collectively, we aimed to identify a new and effective diagnostic tool for CNS involvement in adult patients with AML.

## Methods

### Study population patient cohorts

Ninety AML patients admitted to the First Affiliated Hospital, College of Medicine, Zhejiang University, were enrolled in the study from January 2021 to February 2022. Clinical and laboratory data were collected from the hospital electronic databases. All patients underwent peripheral blood microscopic evaluations, bone marrow and flow cytometric analyses to diagnose AML and classify its morphology. None of the patients had the presence of other diseases that may affect the change of central immune status, such as other tumor, infection, acute trauma to the brain, cerebrovascular disease and a history of immunodeficiency etc. Twenty patients diagnosed with other CNS diseases, such as autoimmune encephalitis (n=6), metastatic brain tumor of lung cancer (n=3), neurosyphilis (n=2), HIV encephalopathy (n=2), viral meningitis (n=3), and headache of unknown cause (n=4), were included for comparison. This study protocol was approved by the ethics committee of the First Affiliated Hospital, College of Medicine, Zhejiang University.

All AML patients underwent diagnostic and follow-up LPs to identify CNS involvement at different time points after their first prophylactic intrathecal chemotherapy therapy with cytarabine (50 mg), dexamethasone (5 mg), and methotrexate 10 mg. To diagnose CNS involvement, we used a cell counting chamber to confirm the presence of >5 white blood cells (WBCs) of CSF. To this end, we also confirmed the presence of leukemic blast cells in the CSF with CC or FCM analysis after cytocentrifugation. Intrathecal therapy for AML in CNS+ patients consisted of intrathecal injection twice a week until CSF blast clearance and minimal residual disease (MRD) FCM were negative.

### CSF samples and cytokine determination

CSF samples (90 CSF samples were obtained at different time points after the first chemotherapy, and 16 CSF samples were obtained after completion of intrathecal prophylactic therapy) were obtained from 90 AML patients and 20 subjects with unrelated CNS involvement. The levels of seven cytokines (IL-2, IL-4, IL-6, and IL-10, tumor necrosis factor-α (TNF-α), interferon-γ (IFN-γ), and IL-17A were quantitatively determined immediately with the use of a cytometric bead array kit (Human Th1/Th2/TH17 Cytokine Kit; JiangXi Cellgene, NanChang, China). The minimum and maximum limits of detection for all 6 cytokines were 0.10 and 5000 pg/ml, respectively. Samples were analyzed using a BD FACS Canto II flow cytometer (Becton Dickinson, San Jose, CA, USA). The data were generated in graphical and tabular format using FCAP Array software (BD Biosciences, San Jose, CA, USA).

### Statistical analysis

Statistical analyses were performed using SPSS version 23.0 (SPSS, Chicago, IL, USA). The Mann–Whitney U test was used to the compare the differences of non-normal distribution data between CNS involvement group and non-CNS involvement group, categorical values were analysed by χ^2^ test or Fisher’s exact test. We evaluated the correlation of clinical features and CSF cytokines with Spearman’s correlation analysis (GraphPad Prism 5.0). The Kolmogorov–Smirnov test on these cytokines revealed a non-normal distribution, thus the Mann–Whitney U test was used to compare the concentrations of seven cytokines among the three groups. The sensitivity, specificity, cut-off value, the area under the curve (AUC), and 95% confidence interval (CI) of CSF cytokine levels to identify CNS involvement was assessed using receiver operating characteristic (ROC) curves (GraphPad Prism 5.0). All tests were two-tailed, and statistical significance was set at p<0.05.

## Results

### Patient characteristics

Ninety adult AML patients and 20 individuals diagnosed with other CNS disease were included in this study (N=110). Patients with AML were divided into those with CNS involvement (CNS+, n=30) and those without CNS involvement (CNS-, n=60). Patients with CNS involvement were significantly older than those without CNS involvement and had a higher proportion of CNS symptoms/signs and adverse cytogenetic risk. Based on the French-American British (FAB) morphology classification scheme, M5 morphologywas associated with CNS involvement. In addition, among the 30 AML patients with CNS+ in this study, 21 cases of FCM of CSF and 18 cases of CC of CSF were positive. Significant differences were observed in white blood cell WBC count, lactate dehydrogenase (LDH) level, CSF protein level, and CSF WBC count, but not in sex, bone marrow status and chemotherapy regimens between the two groups (**Table 1**).

**Table 1:** General characteristic of AML.

Variable	CNS involvement (+)(n=30)	Non-CNS involvement (-)(n=60)	P value
Age, Mean±SD, years	52.21±11.14	44.79±14.06	0.026
Gender, M/F (n)	16/14	32/28	0.56
CNS symptoms/signs	22	4	<0.01
Bone marrow status			
remission	20	43	0.29
no remission	3	5	
relapse	7	12	
cytogenetic risk stratification			<0.01
“Favorable”	5/26	12/53	
“Intermediate”	6/26	29/53	
“Adverse”	15/26	10/53	
FAB classification (n)			
M0	0	0	N/A
M1	1	10	N/A
M2	6	23	<0.01
M3	6	9	0.57
M4	2	3	0.89
M5	15	15	<0.01
M6	0	0	N/A
M7	0	0	N/A
FCM of CSF (n)			N/A
Positive	21	0	
Negative	9	0	
Conventional cytologic of CSF (n)			N/A
Positive	18	0	
Negative	12	0	
Median blood WBCc ×10^9^/L (IQR)	58.21 (6.50-134.58)	7.53 (2.49-31.60)	<0.01
Median lactate dehydrogenase (U/L) (IQR)	664.00(390.50--1761.50)	297.00(216.50-667.50)	<0.01
Median CSF protein (g/L) (IQR)	0.51(0.39-1.09)	0.35(0.29-0.44)	<0.01
Median CSF WBCc×10^6^/L (IQR)	23(2.5-171)	2(1-6)	<0.01
Chemotherapy regimens (n)			0.23
DA-based regimen	7	16	
IA-based regimen	6	14	
HA-based regimen	9	17	
AA-based regimen	4	7	
ATRA-based regimen	4	6	

CNS, central nervous system; SD, standard deviation; WBCc, white blood cell count; IQR, interquartile range; FCM, flowCytometry; CSF, cerebrospinal fluid; N/A, not Applicable; DA-based, Daunorubicin and cytarabine-based; IA-based, idarubicin and cytarabine-based; HA, Homoharringtonine and cytarabine-based; AA, cytarabine+ azacitidine-bsed; ATRA-based, all-trans retinoic acid-based.

### Differences in CSF cytokine concentrations between CNS-positive and CNS-negative patients of the adult AML patients

**Figure 1:**
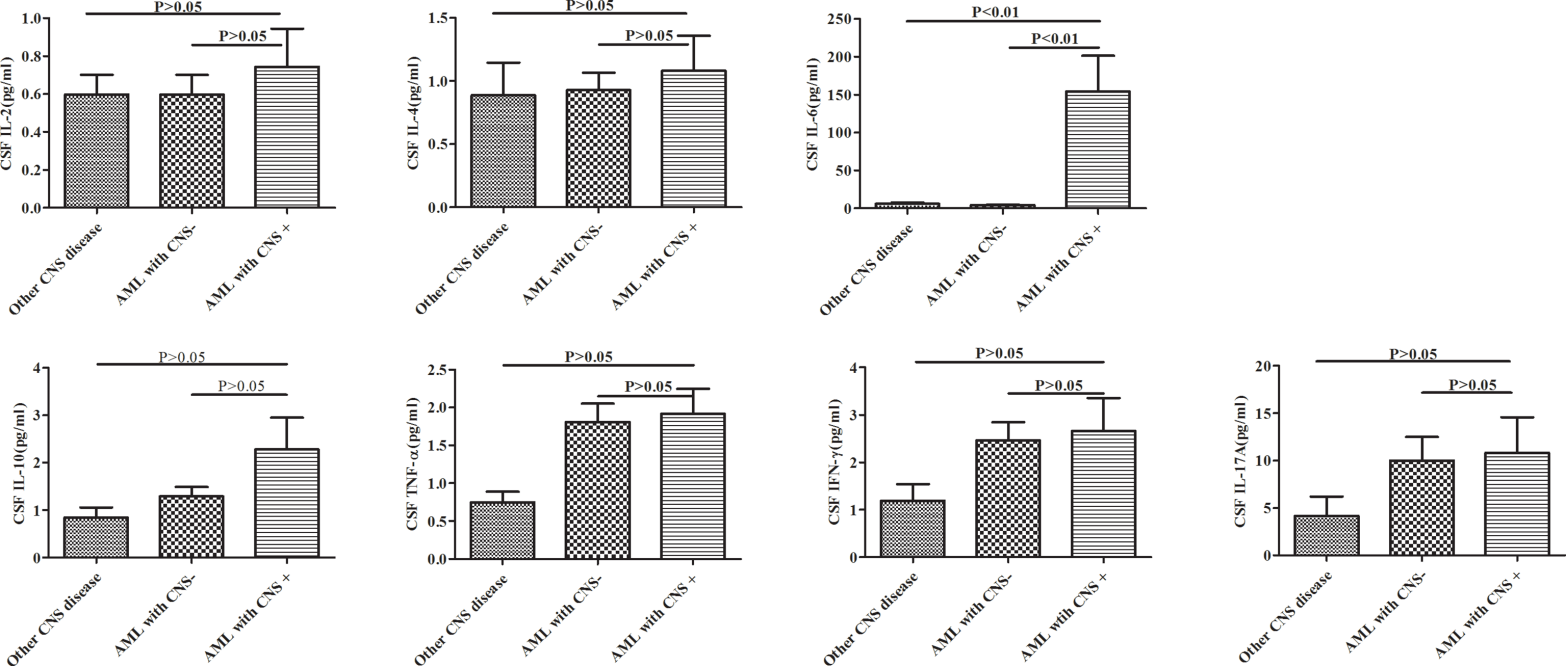
Cytokine profiles in adult AML patients with and without CNS involvement (AML with CNS+ and CNS-) and other CNS diseases. AML, acute myeloid leukemia; CNS, central nervous system.

As shown in **Figure 1**, the CSF IL-6 levels was significantly higher in CNS+ adult AML patients than in the CNS− group and other CNS disease patients (p<0.001). In contrast, no statistically significant differences in the IL-2, IL-4, IL-10, TNF-α, IFN-γ, and IL-17A levels were observed among these groups. At the same time, serum IL-6 results were obtained in 16 CNS+AML patients and 43 CNS-AML patients, but there was no statistically difference between the two groups.(See **Supplementary Figure 1**)

### Correlation between CSF IL-6 level and clinical features

**Figure 2:**
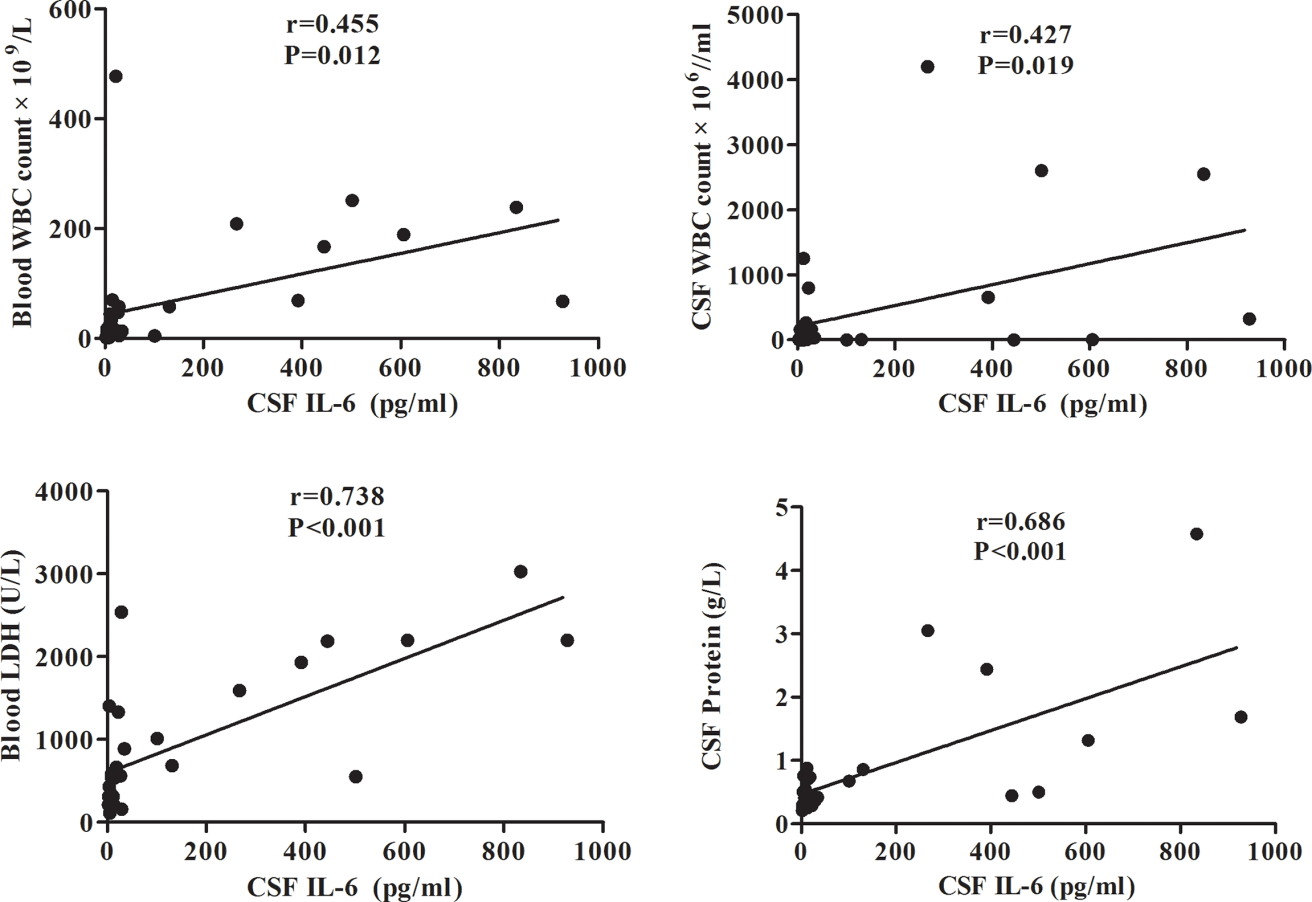
Correlation between the CSF IL-6 level and blood WBC count/CSF WBC count/blood LDH/CSF protein levels in adult AML patients. CSF, cerebrospinal fluid; IL, interleukin; LDH, lactate dehydrogenase; AML, acute myeloid leukemia.

To evaluate the potential correlation between CSF IL-6 levels and clinical features, pairwise Spearman’s rank coefficients were calculated. The data in **Figure 2** suggests that CSF IL-6 levels positively correlated with LDH levels (r=0.738, p<0.001) and blood WBC count (r=0.455, p=0.012), and CSF protein level (r=0.686, p<0.001) and WBC count (r=0.427, p=0.019).

### Diagnosis of CNS involvement based on CSF IL-6 level

**Figure 3:**
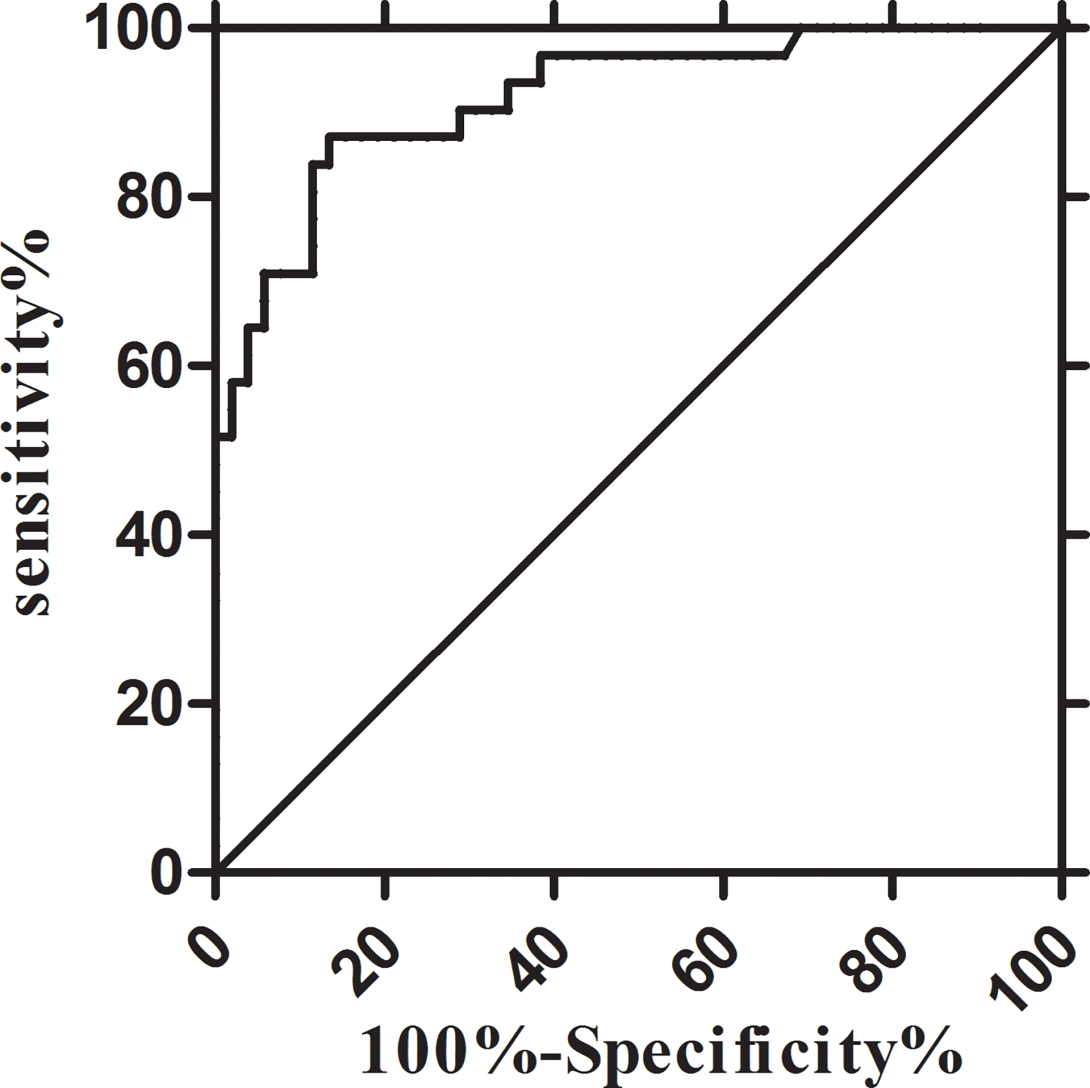
Receiver-operator characteristic (ROC) curves of the CSF IL-6. IL-6: sensitivity 80.00% and specificity 88.46% at 8.27 pg/ml (AUC, 0.8923; 95% CI, 0.8168–0.9678). AUC, area under the curve; CI, confidence interval.

The diagnostic efficacy of CSF IL-6 was assessed using the ROC curve. With the CSF IL-6 cut-off at 8.27 pg/ml, the diagnostic sensitivity and specificity for AML with CNS involvement were 80.00% and 88.46%, respectively (AUC, 0.8923; 95% CI, 0.8168–0.9678) (**Figure 3**). Because the patients with CNS involvement were selected based on positive CC or FCM, the CSF WBC had 100% specificity and discrimination for each procedure. However, the sensitivity of the CC and FCM was only 60.00% and 76.67%, respectively, which was lower than that of CSF IL-6-based diagnosis (**Supplementary Table 1**).

### Correlation between the CSF IL-6 levels and treatment response

**Figure 4:**
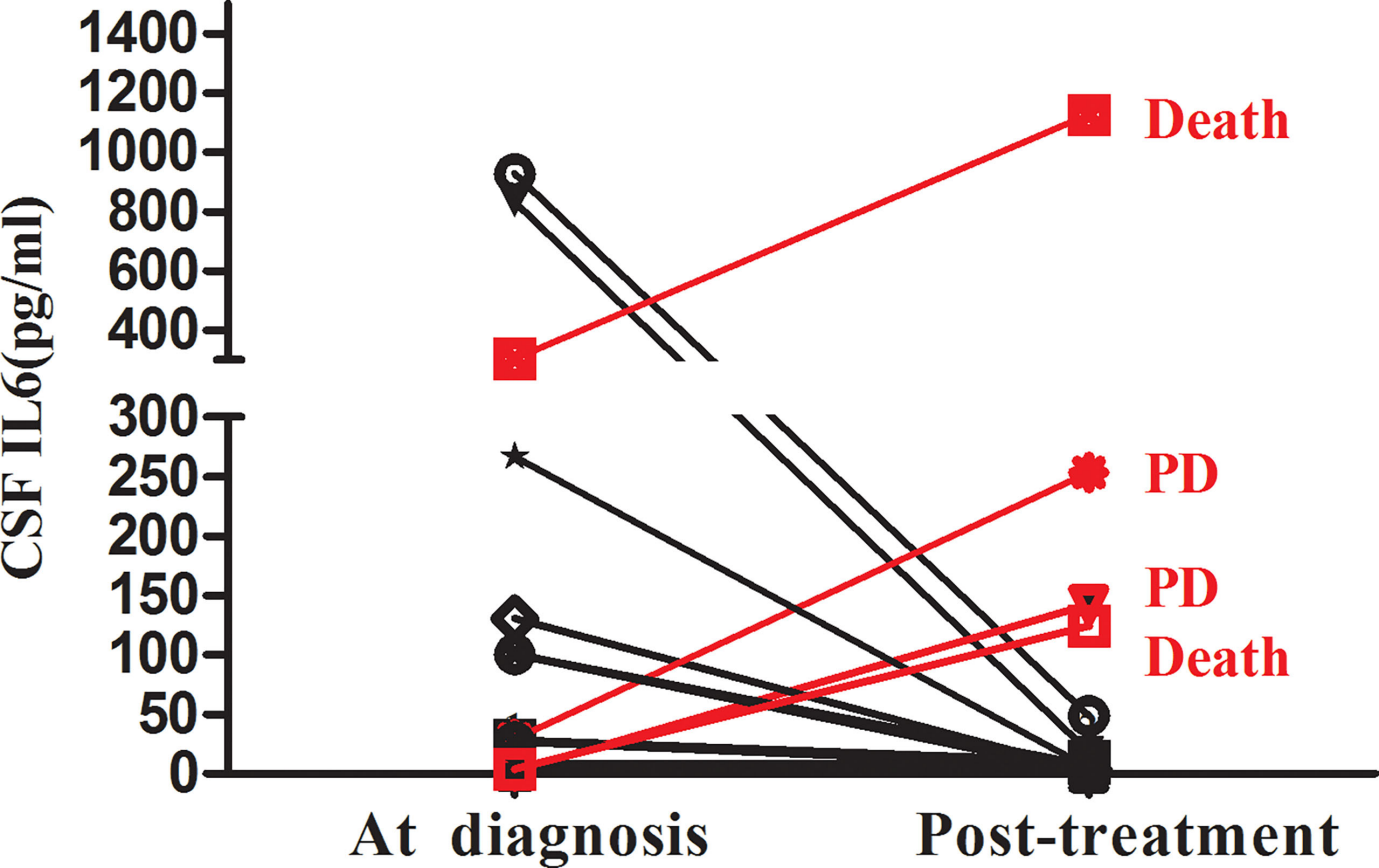
Changes in the CSF IL-6 level between AML with CNS involvement during diagnosis and post-treatment (n=16).

Additionally, we explored the relationship between CSF IL-6 levels and disease status. CSF IL-6 concentrations were available before and after intrathecal treatment in 16 AML patients with CNS involvement. The cytokine levels after chemotherapy treatment decreased in 12 patients, four of whom were in partial remission (PR), whereas the rest were in complete remission (CR). The other four had CSF-IL-6 levels elevation, despite having completed intrathecal chemotherapy treatment; Notably, two of them experienced early death (<12 months), and two reached “progressive disease” status during follow-up visits (**Figure 4**).

## Discussion

CNS involvement in AML remains a clinical challenge related to therapeutic obstacles and confers a poor prognosis (25). In adult AML, occult CNS infiltrations are undetected (3). Moreover, sensitive methods for diagnosing CNS leukemia are still lacking. Cytokines/chemokines involved in tumor metastasis, including leukemia, reportedly play a role in early diagnostics and assessment of metastatic tumor development (26–28). In this retrospective study, we identified a potential biomarker of CNS status in AML. We explored the cytokine concentrations in the CSF of adult patients with AML at CNS relapse. Our results revealed unexpectedly high IL-6 expression levels in CNS+AML patients diagnosed with CC/FCM, whereas those in CNS−AML were similar to those in the control group. We then demonstrated a correlation between CSF IL-6 levels and several clinical features of CNS involvement in adult AML. This is the first demonstration that elevated CSF IL-6 levels have high sensitivity and specificity in diagnosing CNS involvement in adults with AML. Additionally, using serial sample collection in a subset of patients, we examined IL-6 dynamics during treatment and confirmed the significant relationship between the CSF IL-6 levels and the therapeutic response in CNS involvement in adults with AML.

Cytokines are involved in various malignancies. IL-6, a pleiotropic cytokine, plays a role in various cancers, including hematological malignancy (29–31). IL-6 plays a vital role in the cytokines network, which is involved in the regulation of hematopoiesis and leukemic blast formation (32, 33). In patients with AML, serum IL-6 levels have been found to be highly expressed. However, its role as a predictive biomarker or therapeutic target is currently unclear, owing to its contradictory effects (34, 35). Recently, IL-6 has received increasing attention as a vital cytokine involved in tumor invasion (36). In leukemia CNS metastasis, cancerous cells migrate through the vasculature, from the bone marrow to the vertebrae and brain, by crossing the blood-brain-barrier(BBB)via endothelial disruption or trans-endothelial migration, multistep processes (37–41), in which cytokines (such as TNF, IL-1β, or IL-15) and chemokines play critical roles. Moreover, the expression of chemokines and cytokines is positively related to the development of CNS leukemia (28). Here, we revealed that CSF IL-6 concentration was elevated in CNS involvement in adult AML, and the CSF IL-6 levels positively correlated with its clinical features, including elevated blood WBC and LDH levels during diagnosis, as well as CSF protein and WBC, suggesting that IL-6 might play a part in CNS involvement in AML. Furthermore, this novel marker yielded a diagnostic sensitivity of 80.00% and specificity of 88.46%, which provided approximately 20.00% and 10.00% sensitivity benefits, compared to the CC and FCM methods, respectively. As previously reported, CC has a high specificity (>95%), while the sensitivity is relatively low (<50%) (4, 9). In addition, it is difficult to define the precise immunophenotype of neoplastic cells, owing to their paucity (42). In addition, research indicates that IL levels are stable, a significant advantage in contrast to identifying cells within the CSF (20). Thus, our results suggest that CSF IL-6 measurement could be a novel and efficient approach to aid the clinical diagnosis of CNS involvement in adult AML.

However, the cellular origin of CSF IL-6 in this study is unclear. In this study, IL-6 expression in peripheral blood (PB) samples did not differ between AML patients wtih CNS involvement and without CNS involvement, which suggesting that the high expression of CSF IL-6 might not be derived from the peripheral circulation. IL-6 is produced by macrophages (30) as well as neurons, glial and endothelial cells, and fibroblasts, which could play a role in the CNS (43). Thus, we speculated that CSF IL-6 in this study might be produced by non-leukemic cells in the CNS microenvironment. In contrast, a previous study reported that lymphoblasts release multiple cytokines and exosomes and regulate the brain microenvironment to breach the BBB for metastatic invasion (44). Concordantly, emerging evidence demonstrates that cancer cells release cytokines, extracellular vesicles, and exosomes, which have a potential role in altering the microenvironment at extramedullary sites, suggesting that CSF IL-6 is probably to be secreted by leukemia cells (45–47).

Furthermore, we examined the CSF IL-6 levels after intrathecal therapy to evaluate the treatment-induced alteration dynamics of IL-6 expression over time. Our results demonstrated that the CSF IL-6 concentration after treatment was significantly connected with the therapeutic response in adult AML with CNS involvement. Moreover, four patients after intrathecal therapy who were negative for CSF CC/FCM but with an elevated CSF IL-6 concentration exhibited a progressive form of the disease in the subsequent follow-up. Thus, our findings suggest that measurement of IL-6 levels in the CSF could be combined with cytologic/FCM analysis to better assess the therapeutic response as well as to help clinicians determine further treatment. However, whether post-treatment IL-6 levels in the CSF of patients with CR or PR affect PFS requires further investigation.

The present study still had several limitations. First, since CNS involvement is uncommon in adult AML, the sample size was small. Second, as a retrospective study, we lack CSF samples of CNS involvement in adult AML at initial diagnosis; thus, future studies are needed to investigate whether the CSF IL-6 levels in adult AML at initial diagnosis could predict CNS status. Finally, owing to a lack of exploration of the relationship between CSF IL-6 and survival analysis, an accumulation of prospective studies is necessary to provide definitive conclusions on the influence of CSF IL-6 on the prognosis of adult AML with CNS involvement.

## Conclusions

In conclusion, our study provides the first description of CSF IL-6 as a promising diagnostic biomarker for CNS involvement in adult AML patients. We also revealed the correlation between CSF IL-6 level and clinical features of CNS involvement in adult with AML. In addition, we demonstrated that CSF IL-6 level, after intrathecal treatment, correlated with the therapeutic response. Further studies are required to validate CSF IL-6 level as a highly sensitive and specific diagnostic marker for adult AML with CNS involvement in. The molecular mechanisms underlying the cytokine network in the CNS involvement in adult AML require further investigation.

## Data availability statement

The raw data supporting the conclusions of this article will be made available by the authors, without undue reservation.

## Ethics statement

The studies involving human participants were reviewed and approved by Ethics committee of the First Affiliated Hospital, College of Medicine, Zhejiang University. Written informed consent for participation was not required for this study in accordance with the national legislation and the institutional requirements.

## Author contributions

JG and XH was involved in the conception design, analysis and interpretation of the data, and drafting the manuscript. YZ, CB and ZZ collected and analyzed the data and assisted in manuscript drafting. JJ and HT developed the conception design, analyzed and interpreted the data, and revised the manuscript critically for intellectual content. All authors approved the final of the version of the manuscript to be published.

## Conflict of interest

The authors declare that the research was conducted in the absence of any commercial or financial relationships that could be construed as a potential conflict of interest.

## Publisher’s note

All claims expressed in this article are solely those of the authors and do not necessarily represent those of their affiliated organizations, or those of the publisher, the editors and the reviewers. Any product that may be evaluated in this article, or claim that may be made by its manufacturer, is not guaranteed or endorsed by the publisher.
