# Supplementary material for: Cerebrospinal fluid interleukin-6 is a potential diagnostic biomarker for central nervous system involvement in adult acute myeloid leukemia
Source: Front Oncol. 2022 Dec 1;12:1013781. doi: 10.3389/fonc.2022.1013781 (PMC9751393; doi:10.3389/fonc.2022.1013781)
Supplement: Supplementary file 1 [file DataSheet_1.pdf]

Supplementary Material

Biomaker	AUC(%)	SensitivityA(%)	Specificity(%)	PPV(%)	NPV(%)
Conventional cytologic	71.21	60.00	100.00	100	86.11
flow cytometry	86.37	70.00	100.00	100	88.53
CSF IL-6	89.23	80.00	88.46	76.47	92.85

Supplementary table 1 Sensitivities, specificities, positive predictive values and negative predict values of the biomarkers for AML with CNS.

AUC, areas under curve; PPV, positive predictive value; NPV, negative predictive value

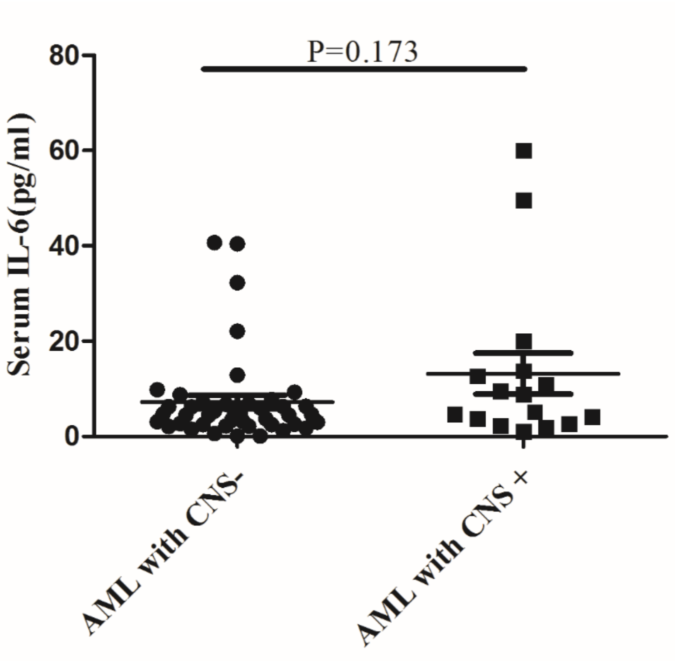

Supplementary Figure 1. Serum IL-6 in adult AML patients with and without CNS involvement (AML with CNS+ and CNS-). AML, acute myeloid leukemia; CNS, central nervous system.
